# Crystal structure of bis­{3-(3,4-di­methyl­phen­yl)-5-[6-(1*H*-pyrazol-1-yl)pyridin-2-yl]-4*H*-1,2,4-triazol-4-ido}iron(II) methanol disolvate

**DOI:** 10.1107/S2056989022009744

**Published:** 2022-10-11

**Authors:** Kateryna Znovjyak, Igor O. Fritsky, Tatiana Y. Sliva, Vladimir M. Amirkhanov, Sergey O. Malinkin, Sergiu Shova, Maksym Seredyuk

**Affiliations:** a Department of Chemistry, Taras Shevchenko National University of Kyiv, Volodymyrska Street 64, Kyiv, 01601, Ukraine; b Department of Inorganic Polymers, "Petru Poni", Institute of Macromolecular Chemistry, Romanian Academy of Science, Aleea Grigore Ghica Voda 41-A, Iasi 700487, Romania; Universität Greifswald, Germany

**Keywords:** crystal structure, spin-crossover, spin transition, energy frameworks

## Abstract

As a result of the high symmetry of the *Aea*2 structure, the asymmetric unit of the title compound, [Fe^II^(C_18_H_15_N_6_)_2_]·2MeOH, consists of half of a charge-neutral complex mol­ecule and a discrete methanol mol­ecule. The planar anionic tridentate ligand 2-[5-(3,4-di­methyl­phen­yl)-4*H*-1,2,4-triazol-3-ato]-6-(1*H*-pyrazol-1-yl)pyridine coordinates the Fe^II^ ion meridionally through the N atoms of the pyrazole, pyridine and triazole groups, forming a pseudo-octa­hedral coordination sphere of the central ion. The average Fe—N bond distance is 1.955 Å, indicating a low-spin state of the Fe^II^ ion. Neighbouring cone-shaped mol­ecules, nested into each other, are linked through double weak C—H(pz)⋯π(ph’) inter­actions into mono-periodic columns, which are further linked through weak C—H⋯N′/C′ inter­actions into di-periodic layers. No inter­actions shorter than the sum of the van der Waals radii of the neighbouring layers are observed. Energy framework analysis at the B3LYP/6–31 G(d,p) theory level, performed to qu­antify the inter­molecular inter­action energies, reproduces the weak inter­layer inter­actions in contrast to the strong inter­action within the layers. Inter­molecular contacts were qu­anti­fied using Hirshfeld surface analysis and two-dimensional fingerprint plots, showing the relative contributions of the contacts to the crystal packing to be H⋯H 48.5%, H⋯C/C⋯H 28.9%, H⋯N/N⋯H 16.2% and C⋯C 2.4%.

## Abstract 2

The title compound, a charge-neutral bis­{2-(3,4-di­methyl­phen­yl)-4*H*-1,2,4-triazol-3-ato)-6-(1*H*-πyrazol-1-yl)pyridine} iron(II) complex di­methanol solvate, is a low-spin complex with a moderately distorted pseudo­octa­hedral coordination environment of the metal ion. As a result of the cone shape, the mol­ecules are stacked in mono-periodic columns that are bound by weak hydrogen bonds into di-periodic layers, which, in turn, are arranged in a three-dimensional lattice bound by weak inter­layer inter­actions.

## 1. Chemical context

Bisazole­pyridines are a broad class of meridional tridentate ligands used to synthesize charged Fe^II^ compounds capable of switching between a spin state with the *t*_2*g*_^4^ *e_g_* ^2^ configuration (high-spin, total spin *S* = 2) and a spin state with the *t*_2*g*_^6^ *e_g_* ^0^ configuration (low-spin, total spin *S* = 0) due to temperature variation, light irradiation or external pressure (Halcrow, 2014 ▸; Halcrow *et al.*, 2019 ▸). In the case of asymmetric ligand design, where one of the azole groups carries a hydrogen on the nitro­gen heteroatom, it was shown that deprotonation can produce neutral complex species that can be high-spin (Schäfer *et al.*, 2013 ▸), low-spin (Shiga *et al.*, 2019 ▸) or exhibit temperature-induced transitions between the spin states of the central atom (Seredyuk *et al.*, 2014 ▸), depending on the ligand field strength. The substituents of ligands can also play an important role in behaviour of the solid samples, determining the way mol­ecules inter­act with each other and, therefore, influencing the spin state adopted by the central atom. As we have recently shown, the dynamic rearrangement of the substituent groups can lead to an abnormally large hysteresis of the thermal high-spin transition due to the supra­molecular mechanism of blocking the deformation of the complex mol­ecule by the meth­oxy group (Seredyuk *et al.*, 2022 ▸). 
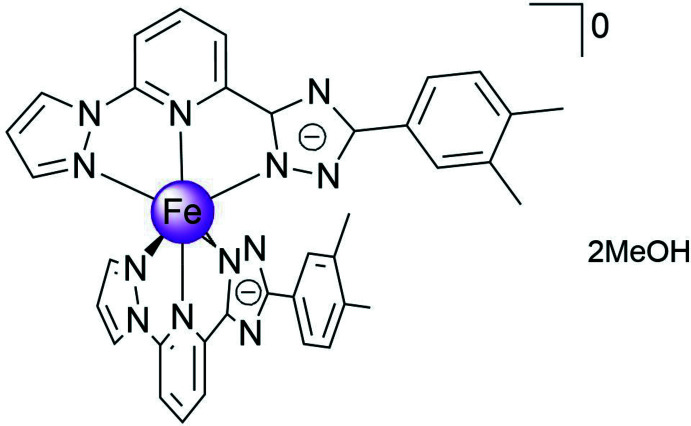


In a continuation of our inter­est in 3*d*-metal complexes formed by polydentate ligands (Bartual-Murgui *et al.*, 2017 ▸; Bonhommeau *et al.*, 2012 ▸; Valverde-Muñoz *et al.*, 2020 ▸), we report here the structural characterization of a new electroneutral complex [Fe^II^ *L*_2_]^0^ based on an asymmetric mono-deprotonated ligand with two methyl substituents on the phenyl group, *L* = 2-[5-(3,4-di­methyl­phen­yl)-4*H*-1,2,4-triazol-3-ato]-6-(1*H*-pyrazol-1-yl)pyridine.

## 2. Structural commentary

**Figure 1:**
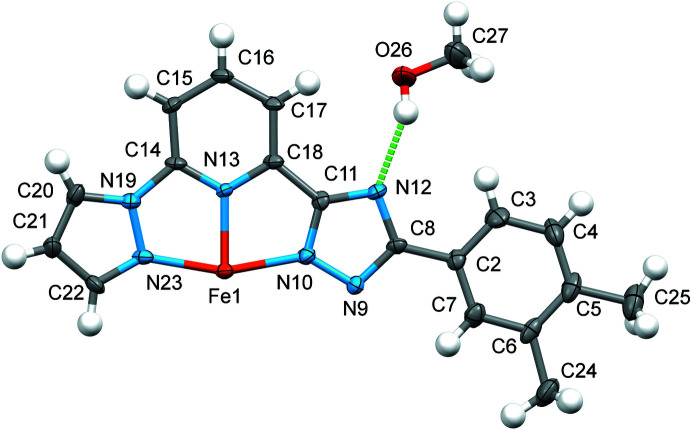
The mol­ecular structure of half the title compound as refined in the asymmetric unit with displacement ellipsoids drawn at the 50% probability level. The O—H⋯N hydrogen bond is indicated by the dashed line. This and the next figure were generated with the program *Mercury* (Macrae *et al.*, 2020 ▸).

The asymmetric unit comprises half of the mol­ecule and a discrete MeOH mol­ecule forming a hydrogen bond O26—H26⋯N12 with the triazole (trz) ring (Fig. 1 ▸). The Fe^II^ ion has a pseudo-octa­hedral coordination environment composed of the nitro­gen donor atoms of the pyrazole (pz), pyridine (py) and trz heterocycles with an average Fe—N distance of 1.957 Å (*V*[FeN_6_] = 9.654 Å^3^) being typical for low-spin complexes with an N_6_ coordination environment (Gütlich & Goodwin, 2004 ▸). The pz, py, trz and phenyl rings, together with the two methyl substituents of one ligand, all lie essentially in the same plane.

The average trigonal distortion parameters, *Σ* = Σ_1_^12^(|90 − *φ*_i_|), with *φ*_i_ being the N—Fe—N′ angle (Drew *et al.*, 1995 ▸), and *Θ* = Σ_1_^24^(|60 − *θ*_i_|), with *θ*_i_ being the angle generated by superposition of two opposite faces of the octa­hedron (Chang *et al.*, 1990 ▸), are 92.8 and 295.0°, respectively. The values reveal a deviation of the coordination environment from an ideal octa­hedron which is, however, in the expected range for complexes with similar bis­azole­pyridine ligands (see below). The calculated continuous shape measure (CShM) value relative to the ideal *O_h_* symmetry is 2.18 (Kershaw Cook *et al.*, 2015 ▸).

## 3. Supra­molecular features

**Figure 2:**
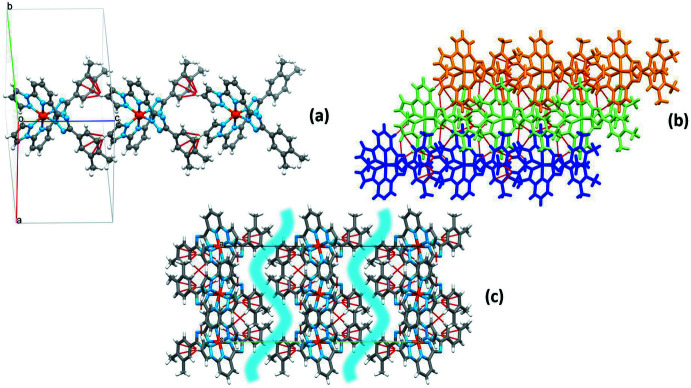
(*a*) A fragment of the mono-periodic supra­molecular columns formed by stacking of mol­ecules along the *c* axis. (*b*) Di-periodic supra­molecular layers formed by stacking of the supra­molecular columns. For a better representation, each column has a different colour. Red dashed lines represent weak hydrogen bonds. (*c*) Stacking of the di-periodic layers along the *c* axis. Blue shaded areas correspond to the inter­layer space without inter­molecular inter­actions shorter than the sum of the van der Waals radii. The methanol mol­ecules are not shown for clarity.

As a result of the tapered shape, neighbouring complex mol­ecules are embedded in each other and inter­act through two weak inter­molecular C—H(pz)⋯π(ph’) contacts between the pyrazole (pz) and phenyl (ph) groups, respectively [distance C2)(pz)⋯*C_g_* (ph’) is 3.392 Å, angle between planes of the rings is 73.77°]. The formed mono-periodic supra­molecular columns protrude along the *c*-axis with a stacking periodicity equal to 10.6511 (7) Å (= cell parameter *c*) (Fig. 2 ▸ *a*)*.* Weak inter­molecular hydrogen-bonding inter­actions C—H(pz, py)⋯N/C(pz, trz)/O(MeOH) in the range 2.257–2.893 Å (Table 1 ▸), link neighbouring columns into corrugated di-periodic layers in the *bc* plane (Fig. 2 ▸ *b*,*c*). The layers stack along the *b*-axis direction without any strong or weak inter­layer inter­actions shorter than the sum of the van der Waals radii (Fig. 2 ▸ *c*). The voids between the layers are occupied by methanol mol­ecules, which participate in the strong hydrogen bonding mentioned above, and weak hydrogen bonding with the aromatic substituents within the layers (a complete list of inter­molecular inter­actions is given in Table 1 ▸).

## 4. Hirshfeld surface and 2D fingerprint plots

**Figure 3:**
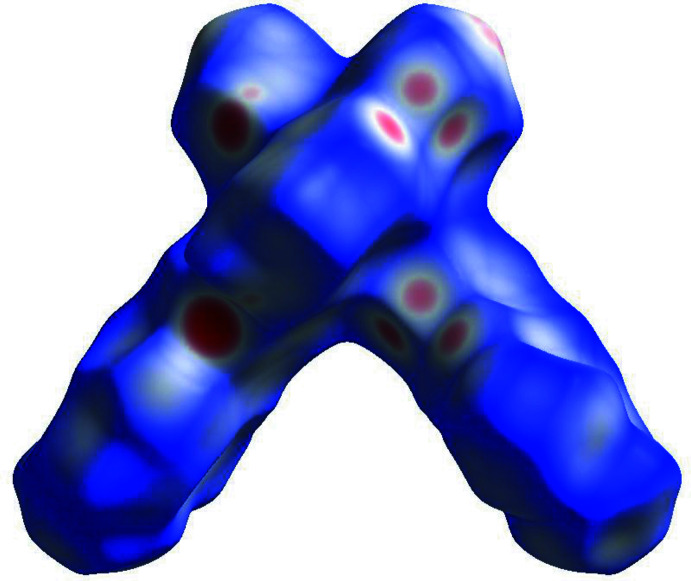
A projection of *d*_norm_ mapped on the Hirshfeld surface, showing the inter­molecular inter­actions within the mol­ecule. Red areas represent regions where contacts are shorter than the sum of the van der Waals radii, blue areas represent regions where contacts are longer than the sum of van der Waals radii, and white areas are regions where contacts are close to the sum of van der Waals radii. This and the next two figures were generated with the program *Crystal Explorer* (Spackman *et al.*, 2021 ▸).

**Figure 4:**
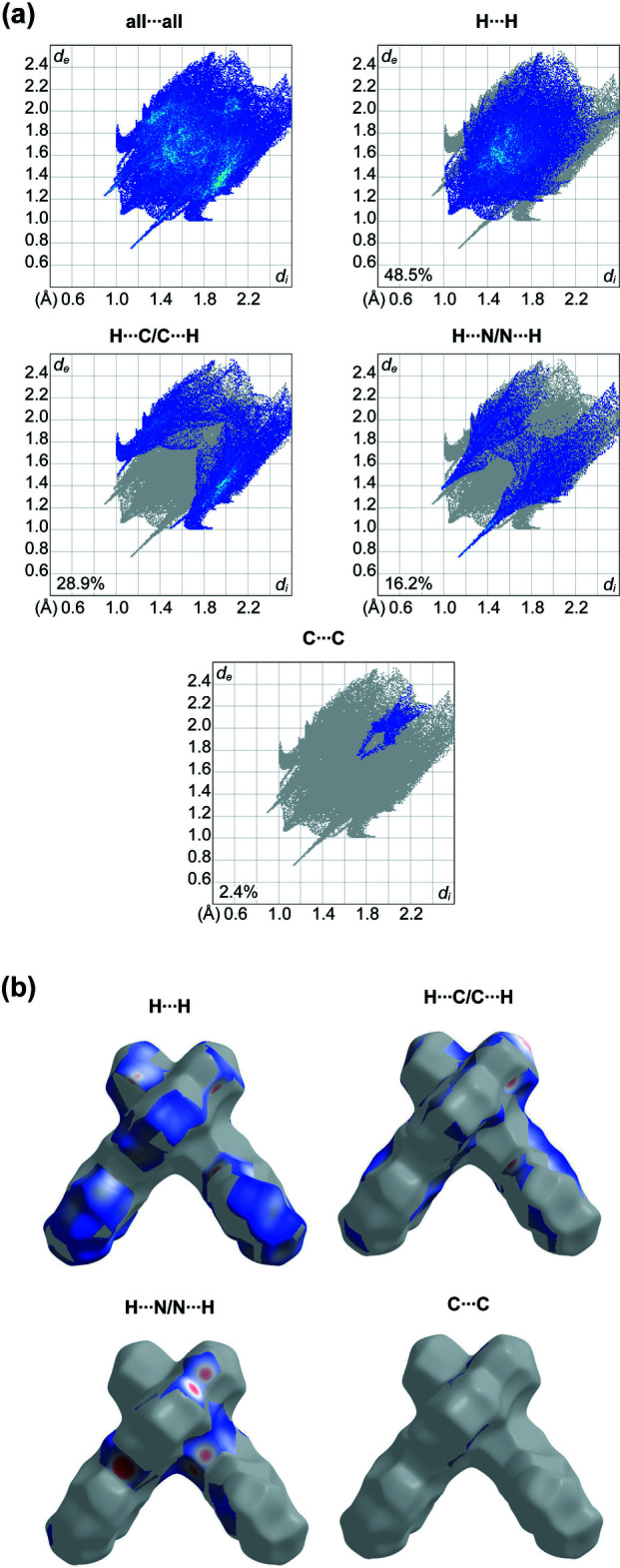
(*a*) The overall two-dimensional fingerprint plot and those decomposed into specified inter­actions. (*b*) Hirshfeld surface representations with the function *d*_norm_ plotted onto the surface for the different inter­actions.

Hirshfeld surface analysis was performed and the associated two-dimensional fingerprint plots were generated using *Crystal Explorer* (Spackman *et al.*, 2021 ▸), with a standard resolution of the three-dimensional *d*_norm_ surfaces plotted over a fixed colour scale of −0.6122 (red) to 1.3609 (blue) a.u. (Fig. 3 ▸). The pale-red spots symbolize short contacts and negative *d*_norm_ values on the surface correspond to the inter­actions described above. The overall two-dimensional fingerprint plot is illustrated in Fig. 4 ▸. The Hirshfeld surfaces mapped over *d*_norm_ are shown for the H⋯H, H⋯C/C⋯H, H⋯N/N⋯H and C⋯C contacts, and the two-dimensional fingerprint plots, associated with their relative contributions to the Hirshfeld surface. At 48.5%, the largest contribution to the overall crystal packing is from H⋯H inter­actions, which are located mostly in the central region of the fingerprint plot. H⋯C/C⋯H contacts contribute 28.9%, resulting in a pair of characteristic wings. The H⋯N/N⋯H contacts, represented by a pair of sharp spikes in the fingerprint plot, make a 16.2% contribution to the Hirshfeld surface. Finally, C⋯C contacts, which account for a contribution of 2.4%, are mostly distributed in the middle part of the plot.

## 5. Energy frameworks

**Figure 5:**
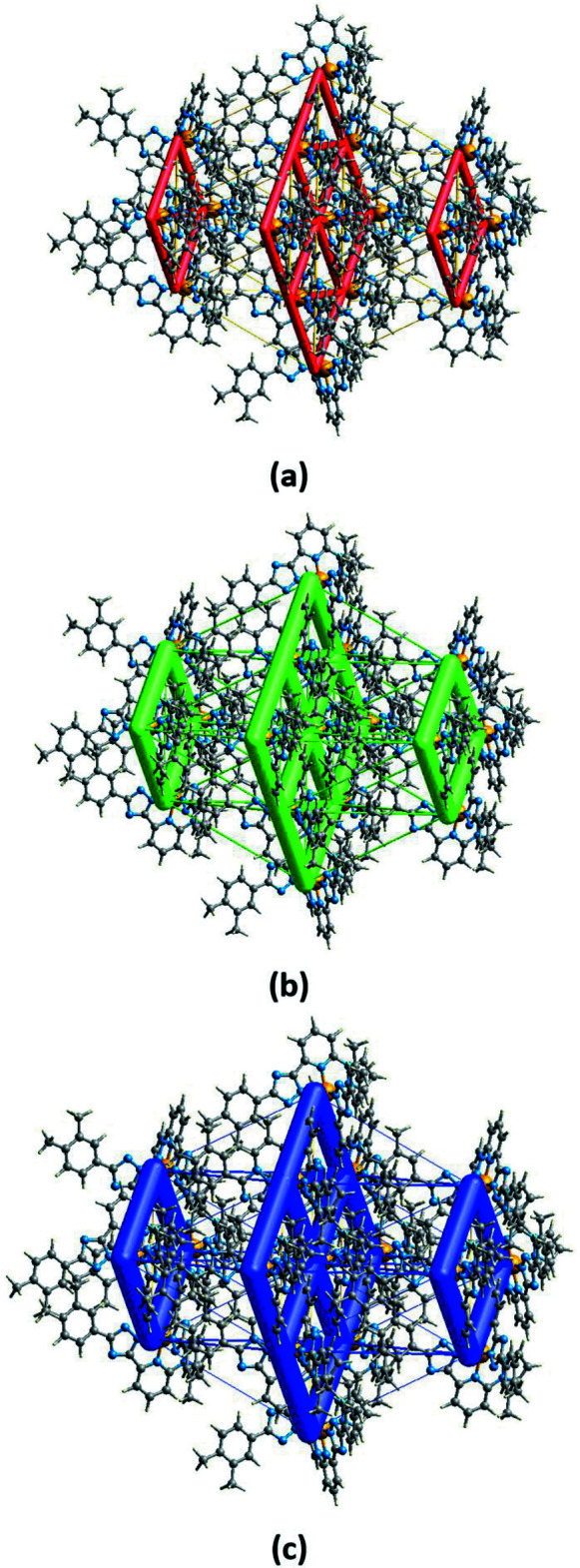
The calculated energy frameworks, showing (*a*) the electrostatic potential forces (*E*_ele_), (*b*) the dispersion forces (*E*_dis_) and (*c*) the total energy diagrams (*E*_tot_). Tube size is set at 100 scale.

The energy frameworks, calculated using the wave function at the B3LYP/6-31G(d,p) theory level, including the electrostatic potential forces (*E*_ele_), the dispersion forces (*E*_dis_) and the total energy diagrams (*E*_tot_), are shown in Fig. 5 ▸ (Spackman *et al.*, 2021 ▸). The cylindrical radii, adjusted to the same scale factor of 100, are proportional to the relative strength of the corresponding energies. The major contribution to the inter­molecular inter­actions comes from dispersion forces (*E*_dis_), reflecting the dominant inter­actions in the network of the electroneutral mol­ecules. The topology of the energy framework resembles the topology of the inter­molecular inter­actions within and between the supra­molecular layers described above. Because of the high lattice symmetry, there are only two different attractive inter­actions between the mol­ecules within the layers, equal to −58.5 and −90.6 kJ mol^−1^. As for the inter­layer inter­actions, the absence of supra­molecular bonding leads to very weak inter­actions in the range −7.4 to +2.5 kJ mol^−1^, *i.e.* from weakly attracting to weakly repulsive. The colour-coded inter­action mappings within a radius of 3.8 Å of a central reference mol­ecule for the title compound together with full details of the various contributions to the total energy (*E*_tot_) are given in the supporting information

## 6. Database survey

A search of the Cambridge Structural Database (CSD, Version 5.42, last update February 2021; Groom *et al.*, 2016 ▸) reveals several similar neutral Fe^II^ complexes with a deprotonated azole group, for example, those based on pyrazole-pyridine-benzimidazole, XODCEB (Shiga *et al.*, 2019 ▸), phenathroline-tetra­zole, QIDJET (Zhang *et al.*, 2007 ▸), and phenanthroline-benzimidazole, DOMQUT (Seredyuk *et al.*, 2014 ▸). We also included in the comparison data for three polymorphs, in different spin states, of a complex structurally similar to the title compound, but carrying a meth­oxy group on the phenyl substituent (EJQOA, BEJQUG, BEJQUG01, BEJRAN, BEJRER; Seredyuk *et al.*, 2022 ▸) (see schematic structures of all complexes in the supporting information. The Fe—N distances of these complexes in the low-spin state are 1.946–1.991 Å, while in the high-spin state they are in the range 2.138–2.184 Å. The values of the trigonal distortion and CShM(*O_h_*) change correspondingly, and in the low-spin state they are systematically lower than in the high-spin state. The respective structural parameters of the title compound and related complexes are given in Table 2 ▸.

## 7. Synthesis and crystallization

The ligand *L* was synthesized by the Suzuki cross-coupling reaction from the commercially available precursors (Enamine Ltd.) according to the method described in the literature (Seredyuk *et al.*, 2022 ▸). The synthesis of the title compound was performed with a layering technique in a standard test tube. The layering sequence was as follows: the bottom layer contained a solution of [Fe(*L*_2_)](BF_4_)_2_ prepared by dissolving *L* = 2-[(3,4-di­methyl­phen­yl)-4*H*-1,2,4-triazol-3-yl)]-6-(1*H*-pyrazol-1-yl)pyridine (100 mg, 0.316 mmol) and Fe(BF_4_)_2_·6H_2_O (53 mg, 0.158 mmol) in boiling acetone, to which chloro­form (5 ml) was then added. The middle layer was a methanol–chloro­form mixture (1:10, 10 ml), which was covered by a layer of methanol (10 ml), to which 100 µl of NEt_3_ was added dropwise. The tube was sealed, and black plate-like single crystals appeared within 3-4 weeks (yield *ca* 75%). Elemental analysis calculated for C_38_H_38_FeN_12_O_2_: C, 60.80; H, 5.10; N, 22.39. Found: C, 60.50; H, 5.31; N, 22.71.

## 8. Refinement

Crystal data, data collection and structure refinement details are summarized in Table 3 ▸. H atoms were placed in calculated positions using idealized geometries, with C—H = 0.98 Å for methyl groups and 0.95 Å for aromatic H atoms, and refined using a riding model with *U*_iso_(H) = 1.2–1.5*U*_eq_(C); the hydrogen atom H26 was refined freely. Two OMIT commands were used to exclude beamstop-affected data.

## Supplementary Material

Crystal structure: contains datablock(s) I. DOI: 10.1107/S2056989022009744/yz2021sup1.cif

Structure factors: contains datablock(s) I. DOI: 10.1107/S2056989022009744/yz2021Isup2.hkl

Supporting information file. DOI: 10.1107/S2056989022009744/yz2021Isup5.cdx

Supporting data for energy framework analysis and schematic structures of related complexes. DOI: 10.1107/S2056989022009744/yz2021sup4.doc

CCDC reference: 2211089

Additional supporting information:  crystallographic information; 3D view; checkCIF report

## Figures and Tables

**Table 1:** Hydrogen bonding (Å) of the title compound

Hydrogen bond	Length	Symmetry operation of the contact atom
C7⋯H—C21(pz)	2.827	1 − *x*, 1 − *y*, 1 + *z*
C6⋯H—C21(pz)	2.777	1 − *x*, 1 − *y*, 1 + *z*
C5⋯H—C21(pz)	2.756	1 − *x*, 1 − *y*, 1 + *z*
C4⋯H—C21(pz)	2.802	1 − *x*, 1 − *y*, 1 + *z*
C3⋯H—C21(pz)	2.893	1 − *x*, 1 − *y*, 1 + *z*
N9⋯H—C15(py)	2.475	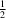 + *x*, 1 − *y*, 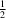 + *z*
N9⋯H—C20(pz)	2.522	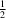 + *x*, 1 − *y*, 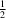 + *z*
H7⋯C20(pz)	2.641	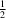 + *x*, 1 − *y*, 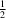 + *z*
N12⋯H—O26	2.017	*x*, *y*, *z*
H17⋯O26	2.329	*x*, *y*, *z*
O26⋯H—C22(pz)	2.257	− 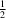 + *x*, 1 − *y*, 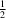 + *z*

**Table 2:** Computed distortion indices (Å, °) for the title compound and similar literature complexes

CSD code	Spin state	<Fe—N>	*Σ*	*Θ*	CShM(*O_h_*)
Title compound	Low-spin	1.957	92.8	295.0	2.18
XODCEB^*a*^	Low-spin	1.950	87.4	276.6	1.92
QIDJET01^*b*^	Low-spin	1.970	90.3	341.3	2.47
QIDJET^*b*^	High-spin	2.184	145.5	553.3	5.88
DOMQIH^*c*^	Low-spin	1.962	83.8	280.7	2.02
DOMQUT^*c*^	Low-spin	1.991	88.5	320.0	2.48
DOMQUT02^*c*^	High-spin	2.183	139.6	486.9	5.31
EJQOA^*d*^	Low-spin	1.946	87.5	308.9	2.16
BEJQUG^*d*^	Low-spin	1.952	97.9	309.9	2.37
BEJQUG01^*d*^	High-spin	2.138	118.0	375.9	3.34
BEJRAN^*d*^	Low-spin	1.946	107.7	384.5	3.20
BEJRE*R* ^*d*^	High-spin	2.139	147.8	507.2	4.92

Notes: (*a*) Shiga *et al.* (2019 ▸); (*b*) Zhang *et al.* (2007 ▸); (*c*) Seredyuk *et al.* (2014 ▸); (*d*) Seredyuk *et al.* (2022 ▸).

**Table 3:** Experimental details

Crystal data	
Chemical formula	[Fe(C_18_H_15_N_6_)_2_]·2CH_4_O
*M*_r_	750.65
Crystal system, space group	Orthorhombic, *A* *e* *a*2
Temperature (K)	180
*a*, *b*, *c* (Å)	12.6854 (10), 26.315 (2), 10.6511 (7)
*V* (Å^3^)	3555.5 (5)
*Z*	4
Radiation type	Mo *K*α
μ (mm^−1^)	0.48
Crystal size (mm)	0.3 × 0.24 × 0.04

Data collection	
Diffractometer	Xcalibur, Eos
Absorption correction	Multi-scan (*CrysAlis PRO*; Rigaku OD, 2022 ▸)
*T*_min_, *T*_max_	0.824, 1.000
No. of measured, independent and observed [*I* > 2σ(*I*)] reflections	6911, 3047, 2211
*R*_int_	0.071
(sin θ/λ)_max_ (Å^−1^)	0.595

Refinement	
*R*[*F* ^2^ > 2σ(*F* ^2^)], *wR*(*F* ^2^), *S*	0.061, 0.100, 1.00
No. of reflections	3047
No. of parameters	247
No. of restraints	1
H-atom treatment	H atoms treated by a mixture of independent and constrained refinement
Δρ_max_, Δρ_min_ (e Å^−3^)	0.84, −0.50
Absolute structure	Flack *x* determined using 703 quotients [(*I* ^+^)−(*I* ^−^)]/[(*I* ^+^)+(*I* ^−^)] (Parsons *et al.*, 2013 ▸).
Absolute structure parameter	−0.02 (3)

Computer programs: *CrysAlis PRO* (Rigaku OD, 2022 ▸), *SHELXT* (Sheldrick, 2015*a* ▸), *SHELXL2018/3* (Sheldrick, 2015*b* ▸) and *OLEX2* (Dolomanov *et al.*, 2009 ▸).

## supplementary crystallographic information

### Crystal data

**Table:**

[Fe(C_18_H_15_N_6_)_2_]·2CH_4_O	*D*_x_ = 1.402 Mg m^−^^3^
*M**_r_* = 750.65	Mo *K*α radiation, λ = 0.71073 Å
Orthorhombic, *A**e**a*2	Cell parameters from 1363 reflections
*a* = 12.6854 (10) Å	θ = 2.6–22.8°
*b* = 26.315 (2) Å	µ = 0.48 mm^−^^1^
*c* = 10.6511 (7) Å	*T* = 180 K
*V* = 3555.5 (5) Å^3^	Plate, clear dark red
*Z* = 4	0.3 × 0.24 × 0.04 mm
*F*(000) = 1568	

### Data collection

**Table:**

Xcalibur, Eos diffractometer	3047 independent reflections
Radiation source: fine-focus sealed X-ray tube, Enhance (Mo) X-ray Source	2211 reflections with *I* > 2σ(*I*)
Graphite monochromator	*R*_int_ = 0.071
Detector resolution: 16.1593 pixels mm^-1^	θ_max_ = 25.0°, θ_min_ = 2.2°
ω scans	*h* = −12→15
Absorption correction: multi-scan (CrysAlisPro; Rigaku OD, 2022)	*k* = −25→31
*T*_min_ = 0.824, *T*_max_ = 1.000	*l* = −12→12
6911 measured reflections	

### Refinement

**Table:**

Refinement on *F*^2^	Hydrogen site location: mixed
Least-squares matrix: full	H atoms treated by a mixture of independent and constrained refinement
*R*[*F*^2^ > 2σ(*F*^2^)] = 0.061	*w* = 1/[σ^2^(*F*_o_^2^) + (0.0192*P*)^2^] where *P* = (*F*_o_^2^ + 2*F*_c_^2^)/3
*wR*(*F*^2^) = 0.100	(Δ/σ)_max_ < 0.001
*S* = 1.00	Δρ_max_ = 0.84 e Å^−^^3^
3047 reflections	Δρ_min_ = −0.50 e Å^−^^3^
247 parameters	Absolute structure: Flack *x* determined using 703 quotients [(*I*^+^)-(*I*^-^)]/[(*I*^+^)+(*I*^-^)] (Parsons *et al.*, 2013).
1 restraint	Absolute structure parameter: −0.02 (3)
Primary atom site location: dual	

### Special details

**Table:**

Geometry. All esds (except the esd in the dihedral angle between two l.s. planes) are estimated using the full covariance matrix. The cell esds are taken into account individually in the estimation of esds in distances, angles and torsion angles; correlations between esds in cell parameters are only used when they are defined by crystal symmetry. An approximate (isotropic) treatment of cell esds is used for estimating esds involving l.s. planes.

### Fractional atomic coordinates and isotropic or equivalent isotropic displacement parameters (Å^2^)

**Table:**

	*x*	*y*	*z*	*U*_iso_*/*U*_eq_	
Fe1	0.500000	0.500000	0.32399 (13)	0.0198 (3)	
N13	0.3533 (4)	0.48261 (19)	0.3181 (6)	0.0170 (12)	
N23	0.4520 (4)	0.5467 (2)	0.1903 (5)	0.0202 (15)	
N19	0.3481 (4)	0.5393 (2)	0.1587 (4)	0.0219 (15)	
N10	0.4953 (5)	0.4455 (3)	0.4514 (5)	0.0211 (15)	
O26	0.1937 (5)	0.3536 (3)	0.6135 (7)	0.074 (3)	
H26	0.255 (9)	0.359 (4)	0.602 (8)	0.10 (4)*	
N12	0.3978 (4)	0.3867 (2)	0.5544 (4)	0.0214 (15)	
N9	0.5624 (4)	0.4206 (2)	0.5293 (5)	0.0205 (15)	
C14	0.2907 (5)	0.5053 (3)	0.2340 (6)	0.0189 (17)	
C16	0.1439 (5)	0.4588 (3)	0.3039 (6)	0.0266 (18)	
H16	0.070880	0.450760	0.300859	0.032*	
C11	0.3991 (6)	0.4244 (3)	0.4689 (6)	0.0187 (17)	
C18	0.3142 (5)	0.4458 (3)	0.3926 (6)	0.0208 (17)	
C17	0.2084 (5)	0.4332 (3)	0.3877 (6)	0.0232 (18)	
H17	0.180469	0.407444	0.440732	0.028*	
C2	0.5444 (6)	0.3525 (3)	0.6874 (6)	0.0221 (17)	
C15	0.1835 (5)	0.4957 (3)	0.2245 (5)	0.0231 (17)	
H15	0.139860	0.513428	0.166841	0.028*	
C20	0.3249 (6)	0.5650 (3)	0.0514 (6)	0.028 (2)	
H20	0.258538	0.565681	0.010235	0.034*	
C7	0.6517 (6)	0.3493 (3)	0.7117 (6)	0.031 (2)	
H7	0.698294	0.368889	0.661329	0.037*	
C21	0.4130 (6)	0.5893 (3)	0.0140 (7)	0.031 (2)	
H21	0.420863	0.610314	−0.057996	0.038*	
C6	0.6947 (6)	0.3191 (3)	0.8056 (7)	0.0300 (19)	
C5	0.6260 (7)	0.2904 (3)	0.8814 (7)	0.036 (2)	
C4	0.5194 (7)	0.2928 (3)	0.8593 (6)	0.040 (2)	
H4	0.473076	0.273418	0.910428	0.048*	
C22	0.4901 (6)	0.5776 (3)	0.1021 (7)	0.0274 (19)	
H22	0.560432	0.589870	0.099718	0.033*	
C24	0.8132 (5)	0.3196 (3)	0.8284 (8)	0.048 (2)	
H24A	0.846198	0.345213	0.774413	0.072*	
H24B	0.842489	0.286043	0.808838	0.072*	
H24C	0.827202	0.327787	0.916573	0.072*	
C8	0.5027 (5)	0.3860 (3)	0.5907 (6)	0.0205 (17)	
C3	0.4769 (6)	0.3232 (3)	0.7632 (6)	0.035 (2)	
H3	0.402940	0.323876	0.749434	0.042*	
C27	0.1677 (7)	0.3185 (4)	0.7045 (8)	0.060 (3)	
H27A	0.208643	0.287319	0.691455	0.089*	
H27B	0.092312	0.310638	0.699199	0.089*	
H27C	0.183692	0.332462	0.787647	0.089*	
C25	0.6686 (7)	0.2584 (3)	0.9889 (7)	0.057 (3)	
H25A	0.722244	0.234986	0.956909	0.085*	
H25B	0.610868	0.238992	1.026777	0.085*	
H25C	0.700073	0.280733	1.052319	0.085*	

### Atomic displacement parameters (Å^2^)

**Table:**

	*U*^11^	*U*^22^	*U*^33^	*U*^12^	*U*^13^	*U*^23^
Fe1	0.0140 (7)	0.0239 (8)	0.0216 (6)	−0.0013 (8)	0.000	0.000
N13	0.010 (3)	0.020 (3)	0.022 (3)	0.000 (3)	−0.003 (3)	0.002 (3)
N23	0.014 (3)	0.028 (4)	0.019 (3)	−0.003 (3)	0.000 (3)	−0.002 (3)
N19	0.016 (3)	0.024 (4)	0.025 (3)	−0.002 (3)	−0.002 (3)	0.002 (3)
N10	0.019 (3)	0.026 (4)	0.018 (3)	0.001 (3)	−0.003 (3)	0.002 (3)
O26	0.024 (4)	0.082 (6)	0.115 (6)	0.009 (4)	0.018 (4)	0.066 (5)
N12	0.013 (3)	0.025 (4)	0.026 (3)	0.000 (3)	−0.004 (3)	0.001 (3)
N9	0.020 (4)	0.024 (4)	0.018 (3)	0.000 (3)	−0.003 (3)	0.004 (3)
C14	0.012 (4)	0.024 (5)	0.021 (4)	−0.007 (4)	0.003 (3)	−0.003 (3)
C16	0.013 (4)	0.034 (5)	0.032 (5)	−0.005 (4)	0.003 (3)	0.000 (4)
C11	0.022 (4)	0.019 (5)	0.015 (4)	−0.001 (4)	−0.004 (3)	−0.002 (3)
C18	0.014 (4)	0.028 (5)	0.020 (3)	−0.002 (4)	0.002 (3)	−0.004 (3)
C17	0.012 (4)	0.032 (5)	0.025 (4)	−0.009 (4)	−0.002 (3)	0.000 (4)
C2	0.025 (4)	0.024 (5)	0.017 (4)	−0.002 (4)	−0.005 (3)	−0.001 (3)
C15	0.015 (4)	0.027 (5)	0.028 (4)	0.001 (4)	−0.007 (3)	0.004 (4)
C20	0.027 (5)	0.036 (5)	0.022 (4)	0.014 (4)	0.001 (3)	0.004 (4)
C7	0.032 (5)	0.032 (5)	0.027 (5)	0.010 (4)	0.003 (4)	0.001 (4)
C21	0.024 (5)	0.035 (6)	0.035 (5)	0.006 (5)	0.004 (4)	0.017 (4)
C6	0.038 (5)	0.024 (4)	0.028 (4)	0.013 (4)	−0.013 (4)	−0.007 (4)
C5	0.051 (6)	0.028 (5)	0.030 (4)	0.006 (5)	−0.019 (4)	0.001 (4)
C4	0.056 (6)	0.033 (5)	0.031 (6)	−0.017 (5)	−0.006 (4)	0.013 (4)
C22	0.025 (5)	0.029 (5)	0.029 (4)	−0.010 (4)	0.006 (4)	0.006 (4)
C24	0.040 (5)	0.060 (6)	0.045 (4)	0.020 (5)	−0.008 (5)	0.012 (6)
C8	0.017 (4)	0.024 (5)	0.021 (4)	0.002 (4)	0.002 (3)	−0.005 (3)
C3	0.029 (5)	0.044 (6)	0.031 (4)	−0.009 (4)	−0.011 (4)	0.001 (4)
C27	0.043 (6)	0.056 (7)	0.080 (6)	0.008 (6)	0.020 (5)	0.025 (6)
C25	0.080 (8)	0.044 (7)	0.047 (5)	0.002 (6)	−0.024 (5)	0.012 (4)

### Geometric parameters (Å, º)

**Table:**

Fe1—N13	1.917 (5)	C2—C8	1.455 (9)
Fe1—N13^i^	1.917 (5)	C2—C3	1.407 (9)
Fe1—N23^i^	1.977 (6)	C15—H15	0.9500
Fe1—N23	1.977 (6)	C20—H20	0.9500
Fe1—N10^i^	1.974 (6)	C20—C21	1.349 (9)
Fe1—N10	1.974 (6)	C7—H7	0.9500
N13—C14	1.338 (8)	C7—C6	1.389 (9)
N13—C18	1.348 (8)	C21—H21	0.9500
N23—N19	1.375 (7)	C21—C22	1.389 (9)
N23—C22	1.333 (9)	C6—C5	1.409 (10)
N19—C14	1.403 (8)	C6—C24	1.522 (9)
N19—C20	1.360 (8)	C5—C4	1.373 (10)
N10—N9	1.358 (8)	C5—C25	1.520 (10)
N10—C11	1.355 (9)	C4—H4	0.9500
O26—H26	0.79 (11)	C4—C3	1.406 (9)
O26—C27	1.380 (9)	C22—H22	0.9500
N12—C11	1.345 (8)	C24—H24A	0.9800
N12—C8	1.386 (8)	C24—H24B	0.9800
N9—C8	1.352 (8)	C24—H24C	0.9800
C14—C15	1.387 (8)	C3—H3	0.9500
C16—H16	0.9500	C27—H27A	0.9800
C16—C17	1.386 (9)	C27—H27B	0.9800
C16—C15	1.383 (9)	C27—H27C	0.9800
C11—C18	1.463 (9)	C25—H25A	0.9800
C18—C17	1.384 (8)	C25—H25B	0.9800
C17—H17	0.9500	C25—H25C	0.9800
C2—C7	1.388 (9)		
			
N13—Fe1—N13^i^	176.2 (4)	C14—C15—H15	121.9
N13—Fe1—N23	80.0 (3)	C16—C15—C14	116.2 (6)
N13—Fe1—N23^i^	97.3 (2)	C16—C15—H15	121.9
N13^i^—Fe1—N23	97.3 (2)	N19—C20—H20	126.1
N13^i^—Fe1—N23^i^	80.0 (3)	C21—C20—N19	107.8 (7)
N13—Fe1—N10	79.6 (3)	C21—C20—H20	126.1
N13—Fe1—N10^i^	103.0 (2)	C2—C7—H7	118.2
N13^i^—Fe1—N10^i^	79.6 (3)	C6—C7—C2	123.7 (7)
N13^i^—Fe1—N10	103.0 (2)	C6—C7—H7	118.2
N23^i^—Fe1—N23	87.9 (3)	C20—C21—H21	127.0
N10^i^—Fe1—N23	93.0 (2)	C20—C21—C22	106.1 (7)
N10—Fe1—N23^i^	93.0 (2)	C22—C21—H21	127.0
N10^i^—Fe1—N23^i^	159.5 (2)	C7—C6—C5	118.4 (7)
N10—Fe1—N23	159.5 (2)	C7—C6—C24	119.9 (7)
N10—Fe1—N10^i^	93.2 (4)	C5—C6—C24	121.6 (7)
C14—N13—Fe1	119.4 (5)	C6—C5—C25	120.6 (7)
C14—N13—C18	119.8 (6)	C4—C5—C6	119.1 (7)
C18—N13—Fe1	120.6 (5)	C4—C5—C25	120.3 (8)
N19—N23—Fe1	112.5 (4)	C5—C4—H4	119.1
C22—N23—Fe1	140.8 (5)	C5—C4—C3	121.9 (7)
C22—N23—N19	105.2 (6)	C3—C4—H4	119.1
N23—N19—C14	116.6 (5)	N23—C22—C21	110.9 (7)
C20—N19—N23	110.0 (6)	N23—C22—H22	124.5
C20—N19—C14	133.2 (6)	C21—C22—H22	124.5
N9—N10—Fe1	138.8 (5)	C6—C24—H24A	109.5
C11—N10—Fe1	114.9 (5)	C6—C24—H24B	109.5
C11—N10—N9	106.3 (6)	C6—C24—H24C	109.5
C27—O26—H26	117 (7)	H24A—C24—H24B	109.5
C11—N12—C8	100.8 (6)	H24A—C24—H24C	109.5
C8—N9—N10	105.7 (5)	H24B—C24—H24C	109.5
N13—C14—N19	111.0 (6)	N12—C8—C2	123.7 (6)
N13—C14—C15	123.3 (6)	N9—C8—N12	113.2 (6)
C15—C14—N19	125.7 (6)	N9—C8—C2	123.1 (6)
C17—C16—H16	119.3	C2—C3—C4	119.8 (7)
C15—C16—H16	119.3	C2—C3—H3	120.1
C15—C16—C17	121.4 (7)	C4—C3—H3	120.1
N10—C11—C18	115.4 (6)	O26—C27—H27A	109.5
N12—C11—N10	114.0 (6)	O26—C27—H27B	109.5
N12—C11—C18	130.6 (7)	O26—C27—H27C	109.5
N13—C18—C11	109.5 (6)	H27A—C27—H27B	109.5
N13—C18—C17	120.5 (6)	H27A—C27—H27C	109.5
C17—C18—C11	130.0 (7)	H27B—C27—H27C	109.5
C16—C17—H17	120.6	C5—C25—H25A	109.5
C18—C17—C16	118.7 (7)	C5—C25—H25B	109.5
C18—C17—H17	120.6	C5—C25—H25C	109.5
C7—C2—C8	121.7 (6)	H25A—C25—H25B	109.5
C7—C2—C3	117.2 (7)	H25A—C25—H25C	109.5
C3—C2—C8	121.1 (6)	H25B—C25—H25C	109.5
			
Fe1—N13—C14—N19	1.3 (8)	C11—N12—C8—C2	177.6 (6)
Fe1—N13—C14—C15	−178.9 (5)	C11—C18—C17—C16	−178.4 (6)
Fe1—N13—C18—C11	−0.5 (8)	C18—N13—C14—N19	−174.9 (6)
Fe1—N13—C18—C17	−179.7 (5)	C18—N13—C14—C15	4.9 (10)
Fe1—N23—N19—C14	7.5 (7)	C17—C16—C15—C14	0.0 (10)
Fe1—N23—N19—C20	−168.3 (4)	C2—C7—C6—C5	−0.6 (11)
Fe1—N23—C22—C21	163.0 (6)	C2—C7—C6—C24	−177.8 (7)
Fe1—N10—N9—C8	−178.8 (5)	C15—C16—C17—C18	1.1 (11)
Fe1—N10—C11—N12	178.9 (5)	C20—N19—C14—N13	168.7 (7)
Fe1—N10—C11—C18	−1.0 (8)	C20—N19—C14—C15	−11.1 (12)
N13—C14—C15—C16	−3.1 (10)	C20—C21—C22—N23	0.6 (9)
N13—C18—C17—C16	0.7 (11)	C7—C2—C8—N12	173.2 (6)
N23—N19—C14—N13	−5.8 (8)	C7—C2—C8—N9	−8.5 (11)
N23—N19—C14—C15	174.4 (6)	C7—C2—C3—C4	0.6 (11)
N23—N19—C20—C21	−0.4 (8)	C7—C6—C5—C4	0.5 (11)
N19—N23—C22—C21	−0.8 (8)	C7—C6—C5—C25	−177.4 (7)
N19—C14—C15—C16	176.7 (6)	C6—C5—C4—C3	0.0 (12)
N19—C20—C21—C22	−0.1 (9)	C5—C4—C3—C2	−0.6 (11)
N10—N9—C8—N12	0.9 (8)	C22—N23—N19—C14	176.5 (6)
N10—N9—C8—C2	−177.5 (6)	C22—N23—N19—C20	0.8 (8)
N10—C11—C18—N13	1.0 (8)	C24—C6—C5—C4	177.7 (7)
N10—C11—C18—C17	−179.9 (7)	C24—C6—C5—C25	−0.3 (12)
N12—C11—C18—N13	−178.9 (7)	C8—N12—C11—N10	0.4 (7)
N12—C11—C18—C17	0.2 (13)	C8—N12—C11—C18	−179.8 (7)
N9—N10—C11—N12	0.2 (8)	C8—C2—C7—C6	177.9 (6)
N9—N10—C11—C18	−179.7 (6)	C8—C2—C3—C4	−177.3 (6)
C14—N13—C18—C11	175.7 (6)	C3—C2—C7—C6	0.0 (11)
C14—N13—C18—C17	−3.6 (10)	C3—C2—C8—N12	−8.9 (11)
C14—N19—C20—C21	−175.2 (7)	C3—C2—C8—N9	169.3 (7)
C11—N10—N9—C8	−0.6 (7)	C25—C5—C4—C3	178.0 (7)
C11—N12—C8—N9	−0.8 (7)		

Symmetry code: (i) −*x*+1, −*y*+1, *z*.
