# Supplementary material for: Crystal structure of bis­{3-(3,4-di­methyl­phen­yl)-5-[6-(1H-pyrazol-1-yl)pyridin-2-yl]-4H-1,2,4-triazol-4-ido}iron(II) methanol disolvate
Source: Acta Crystallogr E Crystallogr Commun. 2022 Oct 11;78(Pt 11):1107–12. doi: 10.1107/S2056989022009744 (PMC9638982; doi:10.1107/S2056989022009744)

**Supporting file S1**

**Energy framework analysis**

The full colour-coded interaction mappings of a central reference molecule for the title compound. Full details of the various contributions to the total energy (*E*_tot_) are also given.


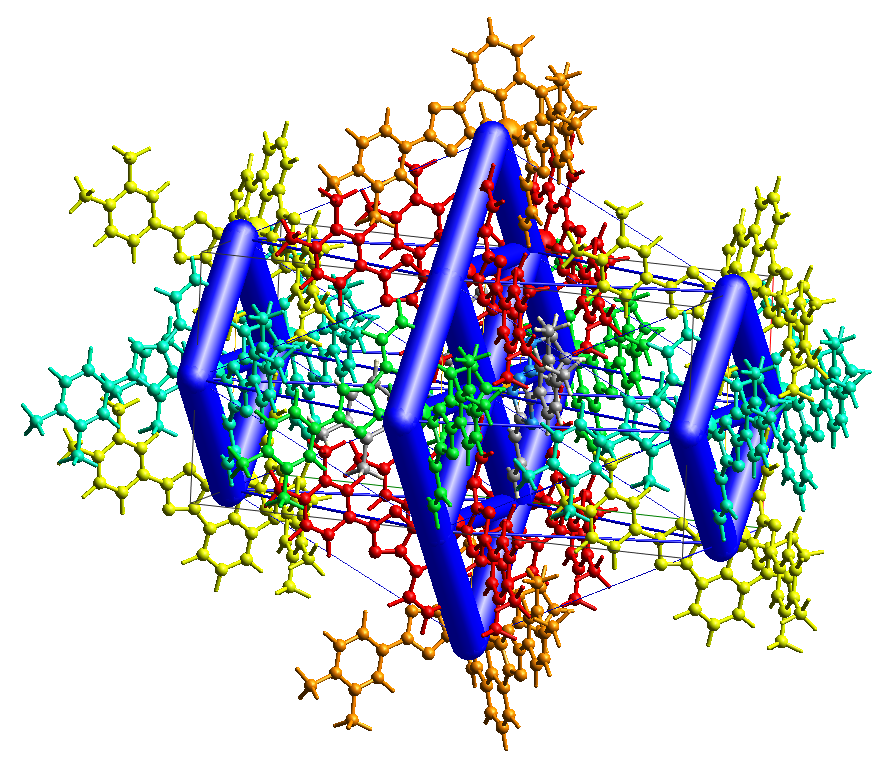


| **Colour code** | **Symmetry opperation** | **R** | ***E*_ele_** | ***E*_pol_** | ***E*_dis_** | ***E*_rep_** | ***E*_tot_** |
| --- | --- | --- | --- | --- | --- | --- | --- |
|  | -x+1/2, y, z+1/2 | 8.28 | -39.7 | -19.0 | -97.2 | 81.1 | -90.6 |
|  | x, y, z | 12.68 | 8.3 | -1.4 | -6.7 | 1.0 | 2.5 |
|  | -x+1/2, y+1/2, z | 14.61 | 2.9 | -0.5 | -8.8 | 4.5 | -2.2 |
|  | x, y, z | 10.65 | -33.4 | -8.8 | -48.2 | 41.0 | -58.5 |
|  | x, y+1/2, z+1/2 | 14.20 | 1.7 | -2.1 | -10.5 | 2.4 | -7.4 |

**Figure** **S1.** Schematic structure of neutral Fe^II^ complexes with deprotonable azol-based ligands.


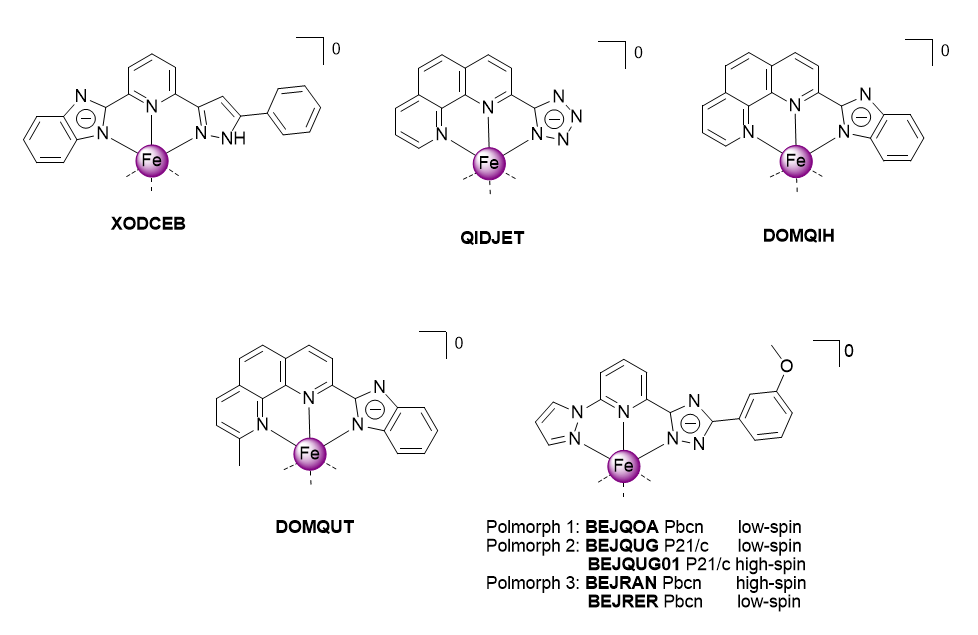

Supplement: Supplementary file 4 [file e-78-01107-sup4.doc]
